# Real‐world experience with eculizumab and switching to ravulizumab for generalized myasthenia gravis

**DOI:** 10.1002/acn3.52051

**Published:** 2024-04-04

**Authors:** Daiki Tokuyasu, Shigeaki Suzuki, Akiyuki Uzawa, Yuriko Nagane, Masayuki Masuda, Shingo Konno, Tomoya Kubota, Makoto Samukawa, Takamichi Sugimoto, Kei Ishizuchi, Munenori Oyama, Manato Yasuda, Hiroyuki Akamine, Yosuke Onishi, Yasushi Suzuki, Naoki Kawaguchi, Naoya Minami, Takashi Kimura, Masanori P. Takahashi, Hiroyuki Murai, Kimiaki Utsugisawa

**Affiliations:** ^1^ Department of Neurology Keio University School of Medicine Tokyo Japan; ^2^ Department of Neurology, Graduate School of Medicine Chiba University Chiba Japan; ^3^ Department of Neurology Hanamaki General Hospital Hanamaki Japan; ^4^ Department of Neurology Tokyo Medical University Tokyo Japan; ^5^ Department of Neurology Toho University Ohashi Medical Center Tokyo Japan; ^6^ Department of Clinical Laboratory and Biomedical Sciences, Division of Health Sciences Osaka University Graduate School of Medicine Osaka Japan; ^7^ Department of Neurology Kindai University Faculty of Medicine Sayama Japan; ^8^ Department of Clinical Neuroscience and Therapeutics Hiroshima University Hiroshima Japan; ^9^ Department of Neurology National Hospital Organization Sendai Medical Center Sendai Japan; ^10^ Department of Neurology Neurology Chiba Clinic Chiba Japan; ^11^ Department of Neurology National Hospital Organization Hokkaido Medical Center Sapporo Japan; ^12^ Department of Neurology Hyogo Medical University Nishinomiya Japan; ^13^ Department of Neurology International University of Health and Welfare Narita Japan

## Abstract

**Objective:**

Eculizumab and ravulizumab are complement protein C5 inhibitors, showing efficacy and tolerability for patients with anti‐acetylcholine receptor‐positive (AChR+) generalized myasthenia gravis (gMG) in phase 3 clinical trials and subsequent analyses. The purpose of the present study was to evaluate the clinical significance of eculizumab and switching to ravulizumab for refractory AChR+ gMG patients in the real‐world experience.

**Methods:**

Among the database of Japan MG registry survey 2021, we studied AChR+ gMG patients who received eculizumab. We also evaluated these patients who switched from eculizumab to ravulizumab. Responder was defined as an improvement of at least 3 points in MG‐ADL. We performed a questionnaire of preference between eculizumab and ravulizumab.

**Results:**

Among 1,106 patients with AChR+ gMG, 36 patients (3%) received eculizumab (female 78%, mean age 56.0 years). Eculizumab was preferentially used in severe and refractory MG patients. The duration of eculizumab treatment was 35 months on average. MG‐ADL improved from 9.4 ± 4.9 to 5.9 ± 5.1, and 25 (70%) of the 36 gMG patients were responders. Postintervention status was markedly improved after the eculizumab treatment. Of 13 patients who did not continue eculizumab, 6 showed insufficiencies. Early onset MG was most effective. However, 15 patients switching from eculizumab to ravulizumab kept favorable response and tolerability. Questionnaire surveys showed preference for ravulizumab over eculizumab.

**Interpretation:**

Eculizumab and switching to ravulizumab showed to be effective for refractory AChR+ gMG patients in clinical settings.

## Introduction

Generalized myasthenia gravis (gMG) is an autoimmune disease that causes severe muscle weakness due to antibody‐mediated damage of the neuromuscular junction.^1^ Most patients with myasthenia gravis have autoantibodies to the acetylcholine receptor (AChR) or muscle‐specific receptor tyrosine kinase.^2^ Treatment for gMG includes corticosteroids, steroid‐sparing immunosuppressive therapies such as calcineurin inhibitors, and fast‐acting treatments (e.g., plasmapheresis, intravenous immunoglobulin, and intravenous methylprednisolone). However, 15–25% of gMG patients who do not respond sufficiently to these therapies, resulting in limited quality of life, exacerbations of MG, hospitalizations, and myasthenic crises are defined as refractory MG.^3^, ^4^

Eculizumab is a humanized monoclonal antibody that binds specifically to human terminal complement protein C5 with high affinity, inhibiting enzymatic cleavage to protein C5a and C5b, and preventing C5a‐induced chemotaxis of proinflammatory cells and formation of C5b‐induced membrane attack complex^5^, ^6^. The REGAIN trial showed the efficacy and tolerability of eculizumab for patients with AChR‐positive (AChR+) refractory gMG.^7^ The REGAIN open‐label extension trial subsequently showed the long‐term efficacy and tolerability of eculizumab.^8^ Although there were additional studies and real‐world data,^9^, ^10^, ^11^, ^12^, ^13^, ^14^ clinical information of gMG patients who did not continue eculizumab was limited. Ravulizumab is a humanized monoclonal antibody partly modified from eculizumab with C5 inhibitory effect in the same way. Ravulizumab has been developed using Xencor's antibody half‐life prolongation technology, which utilizes antibody Fc variants to prolong half‐life.^15^ The CHAMPION study and its extension study also demonstrated the safety and efficacy of ravulizumab in AChR+ gMG patients.^16^, ^17^ However, clinical effectiveness of the switch from eculizumab to ravulizumab in the real‐world experience is not described.

The purposes of the present study were first to evaluate the eculizumab treatment of refractory AChR+ gMG patients for long‐term period and second to report the safety and efficacy of switching to ravulizumab in the real‐world experience.

## Methods

### Patients

**Figure 1:**
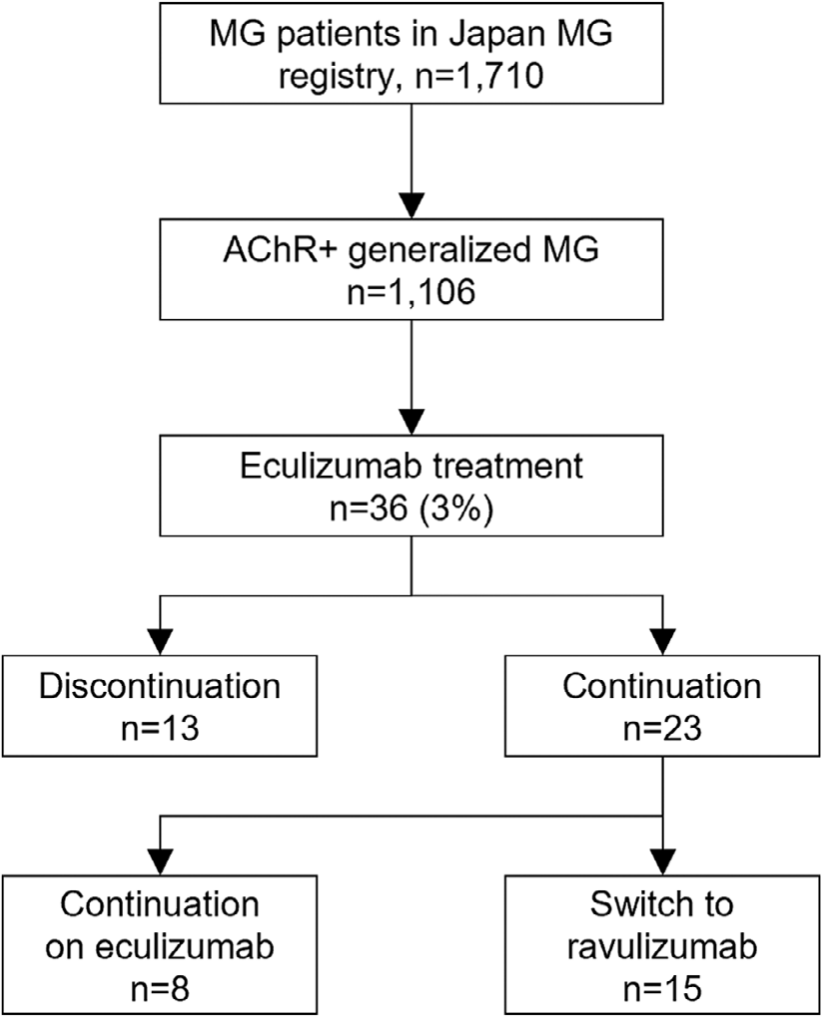
Study flow. AChR+: anti‐acetylcholine receptor‐positive; MG, myasthenia gravis.

The diagnosis of MG was made based on the patient exhibiting the typical history and signs of fluctuating weakness in the voluntary muscles, the presence of serum anti‐AChR or anti‐muscle‐specific receptor tyrosine kinase antibody, and abnormal findings of neuromuscular junction transmission.^1^ Among 1,710 consecutive patients who were followed at 13 institutions joining the Japan MG registry between April and October 2021, there were 1,106 patients with AChR+ generalized MG (Fig. 1).^18^ We evaluated current status, including severity, subtype, and treatment, and retrospectively collected clinical data of onset age, disease duration, sex, past treatment regimens, and disease status at its worst. Early or late‐onset MG is defined as the onset age is <50 years or >50 years. The MG Foundation of America (MGFA) recommendations was used to determine the severity of MG and postintervention status. We collected clinical data during the COVID‐19 pandemic, which limited respiratory test, therefore we focused on only MG activity of daily living (MG‐ADL) for current severity. Refractory MG is defined as occurring in patients whose symptoms cannot be well controlled or who cannot tolerate therapy for adequate control due to side effects and/or burdens even if with ≥2 immunosuppressive oral therapy or with fast‐acting therapy. Responder was defined as an improvement of at least three points in MG‐ADL before and after treatment, as in the REGAIN study.^7^

Eculizumab was approved by the Japanese government for the treatment of AChR+ gMG in December 2017. We included AChR+ gMG patients who received eculizumab between December 2017 and March 2021. Ravulizumab was approved by the Japanese government for the treatment of AChR+ gMG in August 2022. We further studied gMG patients who were switched from eculizumab to ravulizumab until December 2022. At the 26 weeks after ravulizumab treatment corresponding to the fifth infusion, we questionnaire surveyed patients on whether they preferred eculizumab or ravulizumab for 10 items that were used for paroxysmal nocturnal hematuria patients treated with ravulizumab (Table S1).^19^

### Standard protocol approvals, registration, and patient consent

This study was part of the Japan MG registry approved by the ethics committee of each neurological center (Institutional Review Board No. R3‐1 at Hanamaki General Hospital, the primary investigating institute). Written informed consent was obtained from all study participants.

### Statistical analyses

The patients' clinical information was statistically evaluated using SSPS version 29.0.0.0 (IBM SPSS Statistics for Windows). Categorical variables were compared by chi‐squared test. Continuous variables were compared by an analysis of variance (ANOVA) and the Mann–Whitney U test.

## Results

### Clinical features at baseline

Among 1,106 patients with AChR+ gMG enrolled in the Japan MG registry, 36 (3%) were treated with eculizumab. Clinical features of these 36 patients were summarized in comparison to 1,070 patients without eculizumab treatment (Table 1, Table S2). The mean age was 56.0 years, with female predominance (78%). Disease duration before starting eculizumab was 11.3 years on average. Worst MGFA classification showed that all but 1 patients had class III or over, including 12 who experienced a myasthenic crisis. The worst quantitative MG was 21.6 in the entire course of patients with eculizumab treatment, much higher than 14.2 in those without eculizumab treatment (*p* < 0.01). The therapeutic history of the 36 patients showed that oral prednisolone was used in all patients. All but 1 patient also used calcineurin inhibitors. Most patients received fast‐acting treatment. Refractory MG was significantly frequent in the patients with eculizumab more than those without (92% vs. 18%, *p* < 0.01).

**Table 1:** Comparison between AChR+ generalized MG patients with and without eculizumab treatment.

	With eculizumab *n* = 36	Without eculizumab *n* = 1070	*p* value
Age (years)	56.0 ± 17.0	61.7 ± 16.1	0.053
Female	28 (78%)	670 (63%)	0.640
Disease duration (years)	11.3 ± 9.0	12.6 ± 10.7	0.557
MG subtype			
Early onset	13 (36%)	352 (33%)	0.687
Late‐onset	7 (19%)	344 (32%)	0.107
Thymoma‐associated	16 (44%)	374 (35%)	0.241
Worst MGFA classification			
II (mild)	1 (3%)	601 (56%)	<0.01
III (moderate)	13 (36%)	274 (26%)	0.157
IV (severe)	10 (28%)	60 (6%)	<0.01
V (crisis)	12 (33%)	104 (10%)	<0.01
Worst quantitative MG	21.6 ± 8.7	14.2 ± 6.3	<0.01
Immunosuppressive therapy			
Oral prednisolone	36 (100%)	725 (68%)	<0.01
Calcineurin inhibitors	35 (97%)	668 (62%)	<0.01
Thymectomy			
Thymectomy for thymoma	16/16 (100%)	362/365 (99%)	0.716
Thymectomy for non‐thymoma	6/20 (30%)	276/705 (39%)	0.408
Fast‐acting treatment			
Plasmapheresis	34 (94%)	276 (26%)	<0.01
Intravenous immunoglobulin	33 (92%)	305 (29%)	<0.01
Intravenous high‐dose methylprednisolone	35 (97%)	450 (42%)	<0.01
Refractory	33 (92%)	197 (18%)	<0.01

AChR+, anti‐acetylcholine receptor‐positive; MG, myasthenia gravis; MGFA, MG Foundation of America.

### Outcome of eculizumab treatment

**Figure 2:**
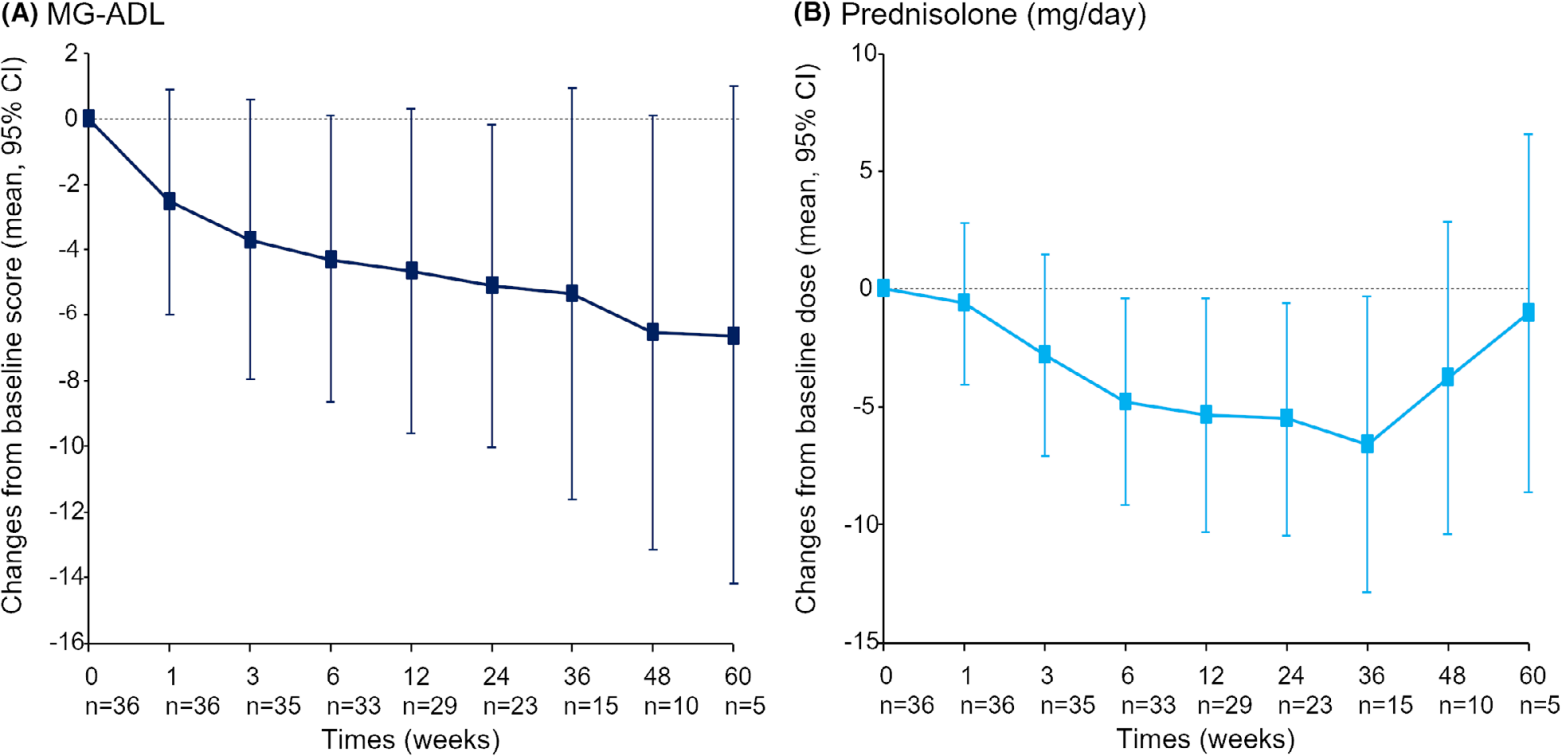
Changes in the mean MG‐ADL (A) and the mean daily prednisolone (B). MG‐ADL, myasthenia gravis activity of daily living.

**Figure 3:**
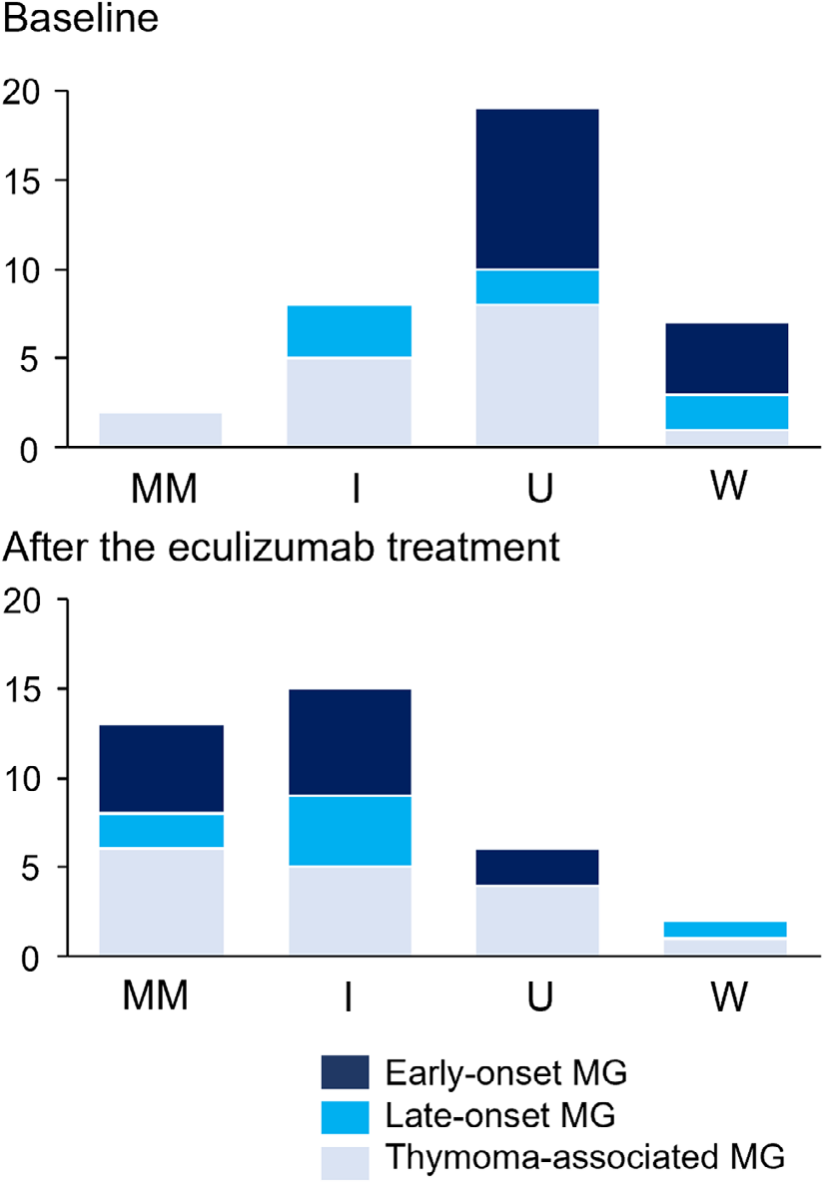
Outcome of patients. Changes in the patients' postintervention status. I, improved; MM, minimal manifestations; U, unchanged; W, worse.

The duration of eculizumab treatment was 35 months in average. The mean MG‐ADL improved from 9.4 ± 4.9 to 5.9 ± 5.1. We showed changes in the mean MG‐ADL in Figure 2A, in which the mean MG‐ADL kept declining over time, although the number of patients was different in each follow‐up period. We found 25 (70%) of the 36 gMG patients treated with eculizumab were responders. There were no significant differences in backgrounds between responders and nonresponders (Table S3). Of the 36 patients, 20 (56%) could reduce the doses of prednisolone. The mean dairy prednisolone dose in the 36 patients was reduced from 15.1 ± 13.3 mg to 11.0 ± 13.1 mg: 12 patients by 1–5 mg, 5 patients by 6–10 mg, and 3 patients by 11 mg or more. We showed changes in the mean daily prednisolone in Figure 2B. The mean daily prednisolone kept declining until 36 weeks, but it re‐increased at 48 and 60 weeks. That is because most of the long follow‐up patients had the same dose of prednisolone as baseline throughout follow‐up periods. Postintervention status was markedly improved after the eculizumab treatment (Fig. 3). At the baseline only 2 patients had minimal manifestations status. After the eculizumab treatment, minimal manifestations increased to 13 patients (36%). Improved status increased from 7 to 15 patients after the eculizumab treatment. However, there were still 8 patients with status unchanged or worse.

### Discontinuation and continuation of eculizumab treatment

Table 2 shows the clinical profiles of the 13 (36%) of 36 gMG patients who did not continue eculizumab. Their duration of eculizumab treatment was 15.5 months in average. The reasons for discontinuation were deaths in 2 patients, severe side effects in 2, minimal manifestations achievements in 2, and inefficacy in 6. In the 2 dead patients, due to stroke and cervical cancer, the responses of eculizumab were favorable. We could not evaluate the efficacy of 2 patients suffering from severe side effects because of the short duration of treatment. Since 3 patients achieved minimal manifestations, the eculizumab treatment was stopped (Fig. S1). In contrast, the duration of eculizumab treatment in the 6 patients who discontinued due to inefficacy was 17 months, ranging from 5 to 39 months. Patients #5 and #9 responded well to eculizumab during 2 years after eculizumab initiation. However, since the efficacy of eculizumab gradually disappeared, both patients stopped the eculizumab treatment (Fig. S2).

**Table 2:** Clinical features of 13 MG patients with a discontinuation of eculizumab therapy.

No./age (years)/sex	Disease duration (months)	MG subset	Worst MGFA classification	Worst quantitative MG	Duration of eculizumab treatment (months)	Causes of discontinuation
#1/50/F	47	EOMG	III	18	12	Death (cervical cancer)
#2/84/F	72	LOMG	III	27	34	Death (cerebral infarction)
#3/32/F	125	EOMG	V	31	1	Adverse effect (dizziness)
#4/75/F	41	LOMG	V	22	3	Adverse effect (severe infection)
#5/37/M	108	TAMG	V	16	33	No effect
#6/41/M	26	TAMG	V	18	13	No effect
#7/47/F	168	TAMG	III	19	6	No effect
#8/48/M	83	TAMG	IV	23	5	No effect
#9/60/F	396	EOMG	II	12	39	No effect
#10/62/F	196	TAMG	IV	20	8	No effect
#11/23/F	89	EOMG	III	14	21	Minimal manifestations
#12/36/F	66	TAMG	III	19	10	Minimal manifestations
#13/62/F	30	TAMG	IV	9	17	Minimal manifestations

EOMG, early onset MG; LOMG, late‐onset MG; MG, myasthenia gravis; MGFA, MG Foundation of America; TAMG, thymoma‐associated MG.

In this study, 23 (64%) of 36 gMG patients continued eculizumab, although there were mild adverse events including infection (*n* = 5) and headache (*n* = 2). Improvement with at least a 3‐point improvement in MG‐ADL was observed in 21 patients. The remaining 2 patients did not show a 3‐point improvement in MG‐ADL, since fast‐acting treatment had been performed immediately before the initiation of eculizumab. Additional treatment to eculizumab was performed in 10 patients including plasmapheresis (*n* = 2), intravenous immunoglobulin (*n* = 5), and intravenous high‐dose methylprednisolone (*n* = 5).

### Comparison among disease subsets

To evaluate differences of effectiveness among disease subsets, we divided the 36 gMG patients with eculizumab treatment into early onset MG (*n* = 13), late‐onset MG (*n* = 7), and thymoma‐associated MG (*n* = 16) (Table 3). There were similar clinical profiles at baseline among 3 subsets. Early‐ onset MG only showed significant improvement; from 11.5 ± 5.7 to 5.2 ± 5.4 in MG‐ADL. In fact, all but one early onset MG patients were responder. In contrast, rates of achievement of minimal manifestations or better status were similar in the 3 subsets.

**Table 3:** Comparison among three disease subsets of MG.

	Early onset MG (*n* = 13)	Late‐onset MG (*n* = 7)	Thymoma‐associated MG (*n* = 16)	*p* value
Age (years)	40.3 ± 15.0	72.9 ± 6.8	53.9 ± 11.4	<0.01
Duration of eculizumab treatment (months)	39.2 ± 26.1	42.7 ± 27.1	27.8 ± 18.3	0.509
Immunosuppressive therapy				
Oral prednisolone	13 (100%)	7 (100%)	16 (100%)	1.000
Calcineurin inhibitors	12 (92%)	6 (86%)	15 (94%)	0.782
Fast‐acting treatment during eculizumab treatment				
Plasmapheresis	1 (8%)	1 (14%)	0	0.302
Intravenous immunoglobulin	4 (31%)	1 (14%)	2 (13%)	0.550
Intravenous high‐dose methylprednisolone	5 (38%)	1 (14%)	1 (6%)	0.084
Reduction of dairy dose of prednisolone (mg)	3.8 ± 4.2	1.7 ± 2.8	4.8 ± 5.5	0.407
MG activity of daily living				
Baseline	11.5 ± 5.7	8.7 ± 4.2	8.2 ± 3.8	0.251
Reduction	6.2 ± 5.0	1.6 ± 4.6	2.1 ± 3.1	0.042
Responders	12 (92%)	4 (57%)	10 (63%)	0.115
Minimal manifestations or better	5 (38%)	2 (29%)	6 (38%)	1.000
Adverse events	3 (23%)	3 (43%)	3 (19%)	0.529
Discontinuation of eculizumab	6 (31%)	2 (29%)	7 (44%)	1.000
Switch to ravulizumab	6 (46%)	4 (57%)	5 (31%)	0.459

MG, myasthenia gravis.

### Switch from eculizumab to ravulizumab

Finally, we evaluated the efficacy and safety of the switch from eculizumab to ravulizumab. There were 15 gMG patients with the switch from eculizumab to ravulizumab (Table S3). MG‐ADL showed 6.2 ± 4.7 at start of ravulizumab and 5.9 ± 5.1 at 26 weeks, respectively. The effect of eculizumab was generally maintained during the ravulizumab treatment since 10 patients showed similar MG‐ADL and postintervention status. Three patients gained three points or over improvement in MG‐ADL including 1 patient newly achieving minimal manifestations. However, 2 patients reported easy fatigability and worsening of MG symptoms during the final 1 week before the following infusion of ravulizumab. In fact, they presented 3‐points or over deterioration in MG‐ADL (from 5 to 8 and from 12 to 17). One of them required intravenous immunoglobulin and intravenous high‐dose methylprednisolone. There were no adverse events, though one patient was lost due to severe COVID‐19 infection.

**Figure 4:**
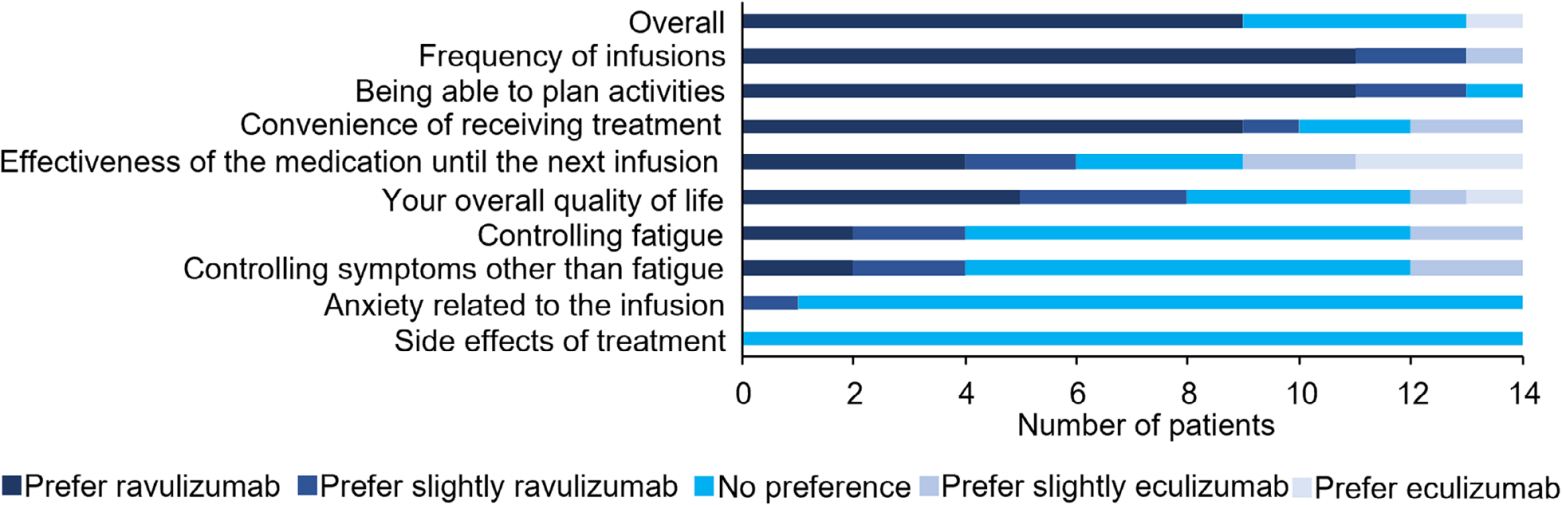
Questionnaire of preferences between eculizumab and ravulizumab.

We further investigated differences between eculizumab and ravulizumab in patients' satisfaction. Figure 4 shows the results of questionnaire surveyed obtained from 14 patients at 26 weeks after the initiation of ravulizumab. Overall, 9/14 (64%) patients preferred ravulizumab more than eculizumab. Especially, ravulizumab was preferred in the items of frequency of infusions, ability to plan activities, the convenience of receiving treatment, and quality of life.

## Discussion

In our real‐world experience, we considered that the efficacy of eculizumab for AChR+ gMG was almost similar to the series of REGAIN.^7^, ^8^, ^9^, ^10^, ^11^, ^12^ At baseline, MG‐ADL were 9.5 in the present study and 10.5 in the REGAIN, respectively.^7^ Reduction in MG‐ADL showed 3.5 in the present study, comparable to the 4.2 in the REGAIN study, although the observation periods were different. Rates of responders were 70% in the present study and up to 84% in the series of REGAIN, respectively.^11^ With regard to the dairy dose of prednisolone, we observed the doses were reduced from 15.1 to 11.0 mg, whereas they were reduced from 17.0 to 12.9 mg in the series of the REGAIN study.^10^ Finally, postintervention status indicated that newly achieved minimal manifestations were observed in 36% of gMG patients in the present study and 51–63% in the series of REGAIN.^12^ The differences may be ascribed to investigators' definition of minimal manifestations because members of the Japan MG registry strictly discussed the status of minimal manifestations.

Eculizumab was administered in only 3% of AChR+ gMG patients, although 5 years had passed since the approval of eculizumab for gMG in Japan. Japanese guidelines for MG recommended that eculizumab should be used only when management of MG is difficult despite intravenous immunoglobulin and plasmapheresis in addition to steroids and immunosuppressive drugs. This refractory MG was estimated to be 21% in the Japan MG registry.^18^ We estimated the limited use of eculizumab was due to not only a potential risk of meningococcal infection but also a financial burden since medical costs of MG are principally covered by the Japanese government. From the view of cost–benefit, the use of eculizumab may be limited in refractory patients or in patients who have failed at least one conventional immunosuppressive therapy^20^, ^21^, and physicians may consider that limited improvements such as two points or less improvement in MG‐ADL does not match the expensive cost of eculizumab. In contrast, it is likely that eculizumab is selected positively for patients with neuromyelitis optica spectrum disorders more than MG since an additional attack has potentially irreversible risks of severe neurological deficits.^22^ Further discussion is needed on which patients should be treated with and continue eculizumab from the view of cost–benefit.

The Asian‐specific C5 gene variant c.2654G > A is a vital mutation that affects eculizumab binding, resulting in nonresponse to eculizumab.^23^ The prevalence of this mutation is reported to be 3.5% in Japanese populations. We thought that six inefficacy patients did not have this mutation based on the data showing suppression of the complement system by blood tests. Although most patients with refractory gMG patients achieved clinical responses by week 12, first responses were also observed with longer‐term treatment.^11^ In the present study, we determined unfavorable responses to eculizumab at least 5 months after treatment. More importantly, 2 patients initially responded to eculizumab during 1–2 years, then but the responses gradually declined.

We thought that the withdrawal of eculizumab was a possible choice when gMG patients could keep minimal manifestations. In the present study, 3 patients stopped eculizumab, however, there was no consensus on how to withdraw eculizumab; reducing doses or expanding an interval of infusions. We believe that the withdrawal of eculizumab can be once considered when gMG patients receive the minimal doses of prednisolone and maintain the state of minimal manifestations for at least 6 months.

Our real‐world experiences also showed that eculizumab treatment elicited sustained improvements across ocular, bulbar, limb, and trunk muscles in refractory gMG patients without meningococcal infections.^9^ To our knowledge, there have been no reports that showed eculizumab was effective particularly in early onset MG compared with late‐onset MG and thymoma‐associated MG. In our real‐world experiences, by comparing MG subsets, eculizumab was significantly effective in the early onset MG more than in the late‐onset MG and thymoma‐associated MG. The reason may be due to that more severe patients were frequently included in the early onset MG. On the other hand, Japanese postmarket surveys revealed that the thymoma‐associated MG showed to be effective in the eculizumab treatment more than the early onset and late‐onset MG.^14^ Younger patients should preferentially receive the benefit of eculizumab therapy from the view of long‐term social contribution and the need of the childcare. There have been case reports of a young woman with gMG treated with eculizumab who was well‐controlled during and after pregnancy.^24^, ^25^

The therapeutic responses were generally maintained after switching to ravulizumab based on MG‐ADL and postintervention status. In addition, tolerability was favorable without severe adverse events, because we thought that 1 death due to COVID‐19 was not related to ravulizumab. Our questionnaire clearly showed patients' satisfaction with ravulizumab compared with eculizumab, resulting in improved quality of life. However, 2 patients noticed easy fatigability and disease fluctuation of MG symptoms when effects of ravulizumab might diminish in an 8‐week interval of injections. In this regard, zilcoplan, a subcutaneously self‐administered macrocyclic peptide inhibitor of C5, is expected to have stable and sustained effects for AChR+ gMG patients.^26^

We had two unavoidable limitations because of observational studies for a longer period. First, there was no strict definition for indication of eculizumab among AChR+ gMG patients as well as adding fast‐acting treatment and reducing doses of prednisolone. Second, the responder was defined by improvement in MG‐ADL, but not in quantitative MG. We utilized quantitative MG only for evaluation of the worst condition during the entire course since COVID‐19 made it difficult to perform respiratory function tests at survey 2021 systematically. In the clinical setting, chronological changes of scoring disease severity of MG are necessary when the initiation or change of molecular‐targeting drugs.

In conclusion, eculizumab and switching to ravulizumab showed to be effective for refractory AChR+ gMG patients in clinical settings.

## Funding Information

This work was supported by the JSPS KAKENHI (grant no. JP20H03592).

## Author Contributions

DT, SS, and KU contributed to the study concept, design, and writing of the manuscript. KI performed statistical analysis. All authors contributed to acquisition of data and analysis, and were involved in drafting the article or critically revising its important intellectual content. They have read and approved the final version of the manuscript.

## Conflict of Interest

D. Tokuyasu declares no potential conflicts of interest related to this article; S. Suzuki received personal fees from Alexion Pharmaceuticals, Argenx, and UCB Pharma, the Japan Blood Products Organization, and Asahi Kasei Medical; A. Uzawa has received honoraria from Alexion Pharmaceuticals and Argenx; Y. Nagane has received speaker honoraria from argenx, Alexion Pharmaceuticals and Japan Blood Products Organization; M. Masuda declares no potential conflicts of interest related to this article; S. Konno declares no potential conflicts of interest related to this article; T. Kubota declares receiving an honorarium for lectures from Alexon Pharmaceuticals, Argenx, and UCB Pharma; M. Samukawa declares no potential conflicts of interest related to this article; T. Sugimoto declares no potential conflicts of interest related to this article; K. Ishizuchi declares no potential conflicts of interest related to this article; M. Oyama declares no potential conflicts of interest related to this article; M. Yasuda declares no potential conflicts of interest related to this article; H. Akamine declares no potential conflicts of interest related to this article; Y. Onishi declares no potential conflicts of interest related to this article; Y. Suzuki declares no potential conflicts of interest related to this article; N. Kawaguchi declares no potential conflicts of interest related to this article; N. Minami declares no potential conflicts of interest related to this article; T. Kimura declares no potential conflicts of interest related to this article; M. P. Takahashi reports unrestricted research grants from Japan Blood Products Organization, Astellas Pharma, Mitsubishi Tanabe Pharma and Pfizer, outside the submitted work, and honorarium for lectures from argenx, Alexion Pharmaceuticals and UCB Pharma; H. Murai has served as a paid consultant for Alexion, AstraZeneca Rare Disease, Argenx, and UCB and has received speaker honoraria from the Japan Blood Products Organization and Chugai Pharmaceutical, and research support from the Ministry of Health, Labour and Welfare, Japan; K. Utsugisawa has served as a paid consultant for UCB Pharma, Argenx, Janssen Pharma, Viela Bio, Chugai Pharma, Hanall BioPharma, and Mitsubishi Tanabe Pharma and has received speaker honoraria from argenx, Alexion Pharmaceuticals, and the Japan Blood Products Organization.

## Supporting information


Figure S1.

Figure S2.

Table S1.

Figure Captions.
