# Supplementary figures and images for: Real‐world experience with eculizumab and switching to ravulizumab for generalized myasthenia gravis
Source: Ann Clin Transl Neurol. 2024 Apr 4;11(5):1338–46. doi: 10.1002/acn3.52051 (PMC11093249; doi:10.1002/acn3.52051)

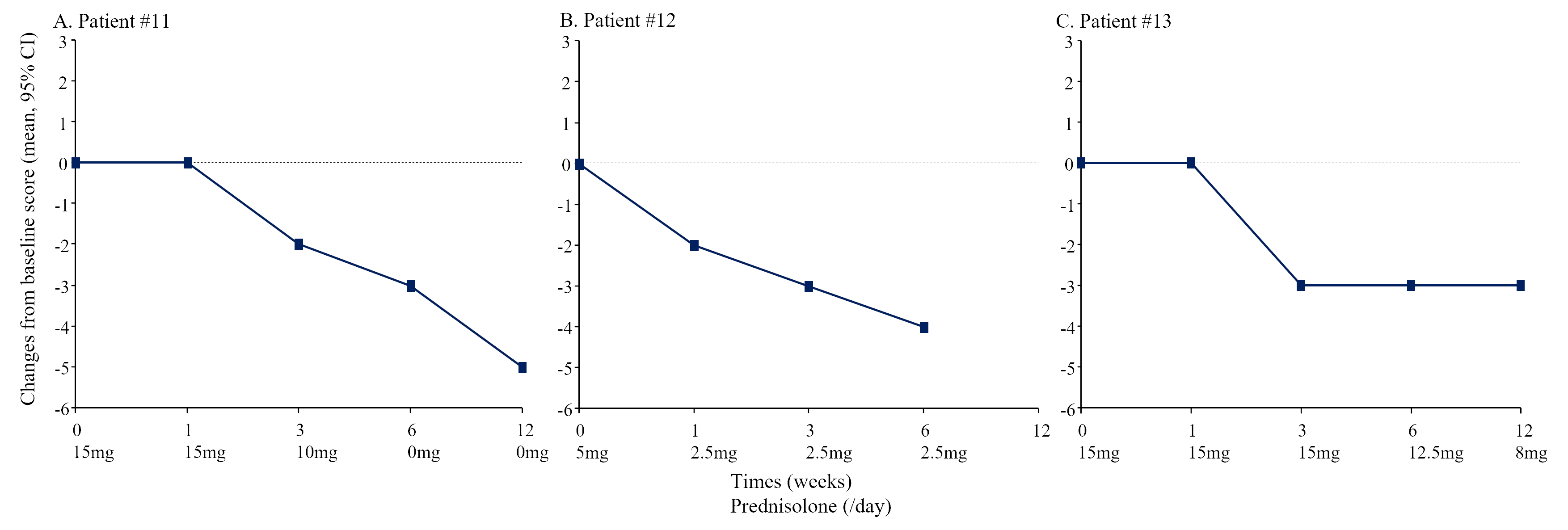

Supplement: Supplementary file 1 — Figure S1. [file ACN3-11-1338-s004.tif]

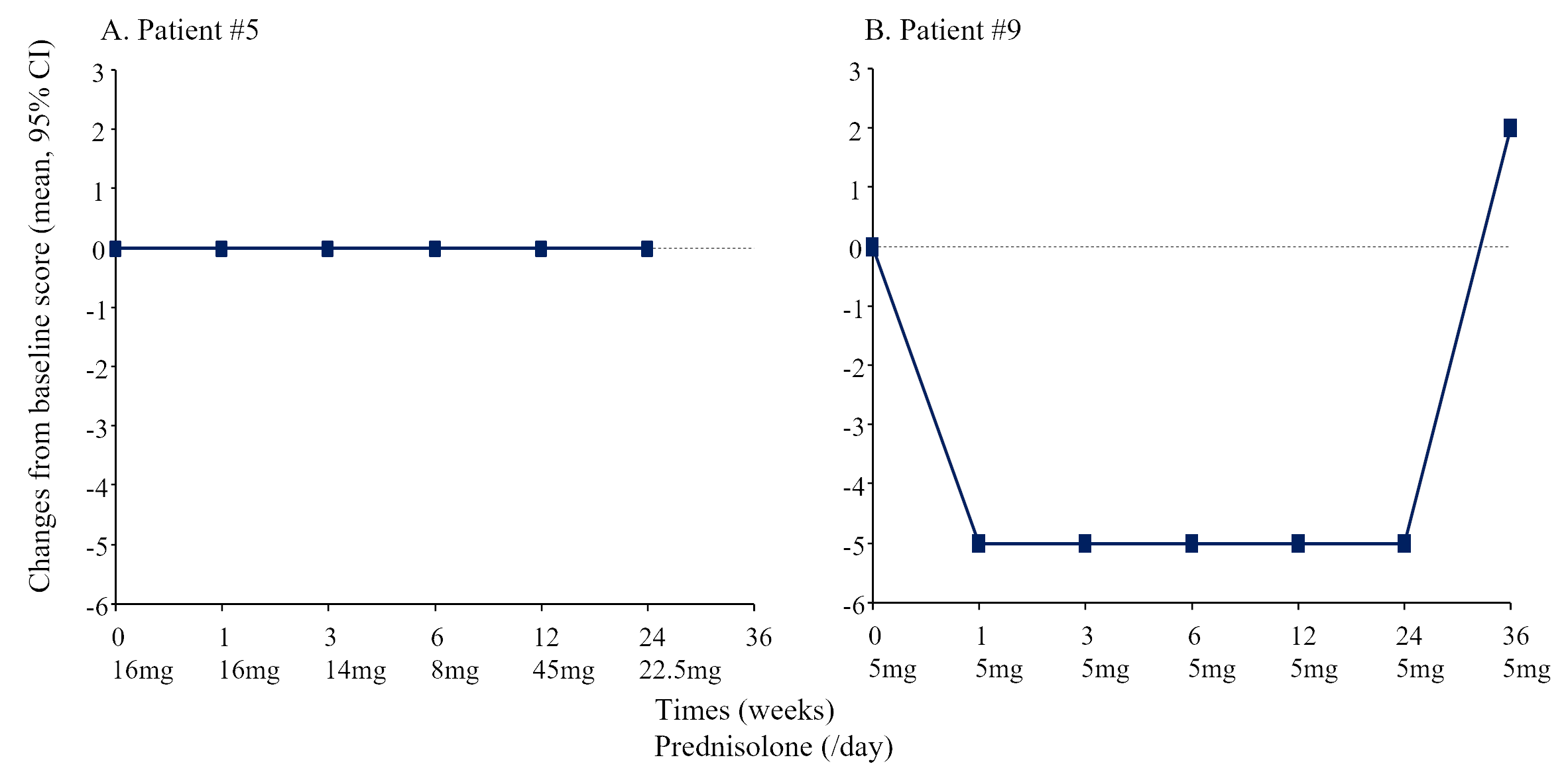

Supplement: Supplementary file 2 — Figure S2. [file ACN3-11-1338-s003.tif]
