# Supplementary material for: Real‐world experience with eculizumab and switching to ravulizumab for generalized myasthenia gravis
Source: Ann Clin Transl Neurol. 2024 Apr 4;11(5):1338–46. doi: 10.1002/acn3.52051 (PMC11093249; doi:10.1002/acn3.52051)
Supplement: Supplementary file 3 — Table S1. [file ACN3-11-1338-s001.docx]

Supplementary Table S1. Form of questionnaire

| **Overall** | | | | |
| --- | --- | --- | --- | --- |
| □Prefer ravulizumab |  | □No preference |  | □Prefer eculizumab |
| **Frequency of infusions** | | | | |
| □Prefer ravulizumab | □Prefer slightly ravulizumab | □No preference | □Prefer slightly eculizumab | □Prefer eculizumab |
| **Being able to plan activities** | | | | |
| □Prefer ravulizumab | □Prefer slightly ravulizumab | □No preference | □Prefer slightly eculizumab | □Prefer eculizumab |
| **Convenience of receiving treatment** | | | | |
| □Prefer ravulizumab | □Prefer slightly ravulizumab | □No preference | □Prefer slightly eculizumab | □Prefer eculizumab |
| **Effectiveness of the medication until the next infusion** | | | | |
| □Prefer ravulizumab | □Prefer slightly ravulizumab | □No preference | □Prefer slightly eculizumab | □Prefer eculizumab |
| **Your overall QoL** | | | | |
| □Prefer ravulizumab | □Prefer slightly ravulizumab | □No preference | □Prefer slightly eculizumab | □Prefer eculizumab |
| **Controlling fatigue** | | | | |
| □Prefer ravulizumab | □Prefer slightly ravulizumab | □No preference | □Prefer slightly eculizumab | □Prefer eculizumab |
| **Controlling symptoms other than fatigue** | | | | |
| □Prefer ravulizumab | □Prefer slightly ravulizumab | □No preference | □Prefer slightly eculizumab | □Prefer eculizumab |
| **Anxiety related to the infusion** | | | | |
| □Prefer ravulizumab | □Prefer slightly ravulizumab | □No preference | □Prefer slightly eculizumab | □Prefer eculizumab |
| **Side effect of treatment** | | | | |
| □Prefer ravulizumab | □Prefer slightly ravulizumab | □No preference | □Prefer slightly eculizumab | □Prefer eculizumab |

Supplementary Table S2. Clinical profiles of 36 MG patients treated with eculizumab.

| No | #1 | #2 | #3 | #4 | #5 | #6 | #7 | #8 | #9 | #10 | #11 | #12 |
| --- | --- | --- | --- | --- | --- | --- | --- | --- | --- | --- | --- | --- |
| Age | 50 | 84 | 32 | 75 | 37 | 41 | 47 | 48 | 60 | 62 | 23 | 36 |
| Sex | Female | Female | Female | Female | Male | Male | Female | Male | Female | Female | Female | Female |
| MG subtype | EOMG | LOMG | EOMG | LOMG | TAMG | TAMG | TAMG | TAMG | EOMG | TAMG | EOMG | TAMG |
| Disease Duration (years) | 5 | 6 | 25 | 5 | 10 | 3 | 14 | 8 | 34 | 3 | 8 | 7 |
| Worst MFGA | 3 | 3 | 5 | 5 | 5 | 5 | 3 | 4 | 2 | 4 | 3 | 3 |
| Worst QMG | 18 | 27 | 31 | 22 | 16 | 18 | 19 | 23 | 12 | 9 | 14 | 19 |
| Thymectomy | No | No | Yes | No | Yes | Yes | Yes | Yes | No | Yes | Yes | Yes |
| Plasmapheresis | No | Yes | Yes | Yes | Yes | Yes | Yes | Yes | Yes | Yes | Yes | Yes |
| IVIg | Yes | Yes | Yes | Yes | Yes | Yes | Yes | Yes | Yes | Yes | Yes | Yes |
| IVMP | Yes | Yes | Yes | Yes | Yes | Yes | Yes | Yes | Yes | Yes | Yes | Yes |
| Refractory | No | Yes | Yes | Yes | Yes | Yes | Yes | Yes | Yes | Yes | No | Yes |
| Duration with eculizumab (months) | 12 | 34 | 1 | 3 | 33 | 13 | 6 | 5 | 39 | 17 | 21 | 10 |
| Baseline |  |  |  |  |  |  |  |  |  |  |  |  |
| MG-ADL | 10 | 4 | 18 | 16 | 2 | 6 | 12 | 15 | 6 | 4 | 5 | 5 |
| Postintervention status | U | W | U | W | I | 1 | U | U | U | I | U | U |
| Prednisolone (mg/day) | 42.5 | 5 | 20 | 15 | 16 | 17.5 | 10 | 16.25 | 5 | 15 | 15 | 5 |
| Calcineurin inhibitors | Cyclosporine 200mg | None | Tacrolimus 3mg | Tacrolimus 3mg | Tacrolimus 3mg | Tacrolimus 3mg | Cyclosporine 150mg | Tacrolimus 3mg | Tacrolimus 3mg | Cyclosporine 200mg | Tacrolimus 3mg | Tacrolimus 3mg |
| After the treatment with eculizumab |  |  |  |  |  |  |  |  |  |  |  |  |
| MG-ADL | 3 | 5 | 18 | 18 | 4 | 9 | 12 | 14 | 1 | 1 | 2 | 2 |
| Post-intervention status | I | I | U | W | I | W | U | U | MM | MM | MM | MM |
| Reduction of prednisolone (mg/day) | 5 | 0 | 0 | 0 | 17 | 15 | 0 | 0 | 0 | 7 | 15 | 0 |
| Prednisolone (mg/day) | 37.5 | 5 | 20 | 15 | 0 | 2.5 | 10 | 16.25 | 5 | 8 | 0 | 5 |
| EFT during the treatment with eculizumab | None | None | None | None | IVIgx9 | None | IVIg | None | IVMP | IVMP | None | None |
| Responder | Yes | No | No | No | No | No | No | No | Yes | Yes | Yes | Yes |
| Continuation | No | No | No | No | No | No | No | No | No | No | No | No |
| Adverse events | None | Meningitis | Dizziness | Infection | None | None | Headache | None | None | None | None | None |
| Switch to ravulizumab | No | No | No | No | No | No | No | No | No | No | No | No |

| No | #13 | #14 | #15 | #16 | #17 | #18 | #19 | #20 | #21 | #22 | #23 | #24 |
| --- | --- | --- | --- | --- | --- | --- | --- | --- | --- | --- | --- | --- |
| Age | 62 | 22 | 22 | 27 | 33 | 35 | 40 | 46 | 51 | 51 | 54 | 54 |
| Sex | Female | Female | Female | Female | Female | Male | Male | Male | Female | Female | Male | Female |
| MG subtype | TAMG | EOMG | EOMG | EOMG | EOMG | EOMG | TAMG | EOMG | TAMG | EOMG | TAMG | EOMG |
| Disease Duration (years) | 17 | 10 | 6 | 6 | 6 | 9 | 8 | 8 | 21 | 3 | 9 | 17 |
| Worst MFGA | 4 | 5 | 5 | 5 | 3 | 3 | 4 | 3 | 5 | 4 | 3 | 4 |
| Worst QMG | 20 | 24 | 37 | 39 | 17 | 20 | 14 | 13 | 30 | 30 | 7 | 27 |
| Thymectomy | Yes | Yes | No | Yes | No | No | Yes | No | Yes | Yes | Yes | No |
| Plasmapheresis | Yes | Yes | Yes | Yes | Yes | Yes | Yes | Yes | Yes | Yes | Yes | Yes |
| IVIg | Yes | Yes | Yes | Yes | Yes | No | Yes | Yes | No | Yes | Yes | Yes |
| IVMP | Yes | Yes | Yes | Yes | Yes | No | Yes | Yes | Yes | Yes | Yes | Yes |
| Refractory | Yes | Yes | Yes | Yes | Yes | Yes | Yes | Yes | Yes | Yes | Yes | Yes |
| Duration with eculizumab (months) | 8 | 42 | 56 | 23 | 51 | 29 | 46 | 32 | 58 | 23 | 9 | 96 |
| Baseline |  |  |  |  |  |  |  |  |  |  |  |  |
| MG-ADL | 8 | 18 | 17 | 1 | 17 | 13 | 11 | 6 | 15 | 10 | 6 | 19 |
| Postintervention status | U | W | W | U | U | W | W | U | U | U | I | W |
| Prednisolone (mg/day) | 5 | 20 | 24 | 13 | 17 | 5 | 70 | 10 | 7.5 | 50 | 12.5 | 10 |
| Calcineurin inhibitors | Cyclosporine 150mg | Tacrolimus 3mg | None | Tacrolimus 3mg | Cyclosporine 250mg | Tacrolimus 3mg | None | Tacrolimus 3mg | Tacrolimus 2mg | Tacrolimus 3mg | Tacrolimus 2mg | Cyclosporine 250mg |
| After the treatment with eculizumab |  |  |  |  |  |  |  |  |  |  |  |  |
| MG-ADL | 10 | 12 | 13 | 0 | 5 | 1 | 4 | 6 | 15 | 4 | 3 | 1 |
| Post-intervention status | U | I | I | MM | I | MM | I | U | U | I | I | MM |
| Reduction of prednisolone (mg/day) | 0 | 0 | 8 | 0 | 5 | 1 | 5 | 5 | 5 | 5 | 10 | 5 |
| Prednisolone (mg/day) | 5 | 20 | 16 | 13 | 12 | 4 | 65 | 5 | 2.5 | 45 | 2.5 | 5 |
| EFT during the treatment with eculizumab | None | IVIgx2 | PE | IVMP. IVIg, PE | IVIg | None | None | IVIgx3 | None | IVMP | None | IVMP |
| Responder | No | Yes | Yes | Yes | Yes | Yes | Yes | Yes | No | Yes | Yes | Yes |
| Continuation | No | No | Yes | No | Yes | No | No | No | No | No | No | Yes |
| Adverse events | Infection | Headache | None | None | None | None | None | Infection | None | None | None | None |
| Switch to ravulizumab | No | Yes | No | Yes | No | Yes | Yes | Yes | Yes | Yes | Yes | No |

| No | #25 | #26 | #27 | #28 | #29 | #30 | #31 | #32 | #33 | #34 | #35 | #36 |
| --- | --- | --- | --- | --- | --- | --- | --- | --- | --- | --- | --- | --- |
| Age | 55 | 57 | 61 | 64 | 66 | 67 | 68 | 69 | 69 | 74 | 76 | 78 |
| Sex | Male | Female | Female | Female | Female | Female | Female | Female | Female | Female | Female | Female |
| MG subtype | TAMG | TAMG | TAMG | LOMG | LOMG | LOMG | TAMG | EOMG | TAMG | TAMG | LOMG | LOMG |
| Disease Duration (years) | 15 | 24 | 16 | 7 | 22 | 15 | 4 | 29 | 18 | 43 | 5 | 7 |
| Worst MFGA | 5 | 5 | 4 | 3 | 3 | 5 | 4 | 3 | 5 | 4 | 4 | 3 |
| Worst QMG | 39 | 24 | 23 | 12 | 14 | 32 | 22 | 14 | 39 | 26 | 16 | 10 |
| Thymectomy | Yes | Yes | Yes | No | No | Yes | Yes | Yes | Yes | Yes | No | No |
| Plasmapheresis | Yes | Yes | Yes | Yes | Yes | Yes | Yes | Yes | Yes | Yes | No | Yes |
| IVIg | No | Yes | Yes | Yes | Yes | Yes | Yes | Yes | Yes | Yes | Yes | Yes |
| IVMP | Yes | Yes | Yes | Yes | Yes | Yes | Yes | Yes | Yes | Yes | Yes | Yes |
| Refractory | Yes | Yes | Yes | Yes | No | Yes | Yes | Yes | Yes | Yes | Yes | Yes |
| Duration with eculizumab (months) | 60 | 31 | 35 | 30 | 84 | 79 | 31 | 84 | 52 | 30 | 44 | 25 |
| Baseline |  |  |  |  |  |  |  |  |  |  |  |  |
| MG-ADL | 6 | 11 | 12 | 5 | 12 | 6 | 5 | 9 | 7 | 6 | 12 | 6 |
| Postintervention status | MM | U | U | I | U | I | I | U | MM | U | U | I |
| Prednisolone (mg/day) | 5 | 15 | 8 | 12.5 | 5 | 10 | 20 | 5 | 9 | 10 | 10 | 8 |
| Calcineurin inhibitors | Tacrolimus 3mg | Tacrolimus 3mg | Tacrolimus 3mg | Tacrolimus 3mg | Cyclosporine 100mg | Cyclosporine 100mg | Tacrolimus 3mg | Cyclosporine 125mg | Tacrolimus 5mg | Cyclosporine 100mg | Tacrolimus 1mg | Tacrolimus 1mg |
| After the treatment with eculizumab |  |  |  |  |  |  |  |  |  |  |  |  |
| MG-ADL | 3 | 2 | 8 | 0 | 2 | 11 | 1 | 2 | 4 | 5 | 9 | 5 |
| Post-intervention status | MM | MM | I | MM | MM | I | MM | I | MM | I | I | I |
| Reduction of prednisolone (mg/day) | 0 | 10 | 5 | 5 | 0 | 0 | 3 | 0 | 5 | 5 | 0 | 7 |
| Prednisolone (mg/day) | 5 | 5 | 3 | 7.5 | 5 | 10 | 17 | 5 | 4 | 5 | 10 | 1 |
| EFT during the treatment with eculizumab | None | None | None | None | None | IAPP | None | IVMP | None | None | IVMP, IVIg | None |
| Responder | Yes | Yes | Yes | Yes | Yes | No | Yes | Yes | Yes | Yes | Yes | Yes |
| Continuation | Yes | No | Yes | Yes | No | No | Yes | No | Yes | No | No | No |
| Adverse events | None | None | None | None | None | None | None | None | None | COVID19 | None | Infection |
| Switch to ravulizumab | No | Yes | No | No | Yes | Yes | No | Yes | No | Yes | Yes | Yes |

Supplementary Table S3. Comparison of backgrounds between responders and non-responders.

|  | Responders  n=26 | Non-responders  n=10 | P-values |
| --- | --- | --- | --- |
| Age (y) | 55.2±17.3 | 58.0±16.2 | 0.667 |
| Female | 21 (81%) | 7 (70%) | 0.639 |
| Disease duration (y) | 12.7±10.0 | 12.4±6.9 | 0.934 |
| MG subtype  　Early-onset  　Late-onset  　Thymoma-associated | 12 (46%)  4 (15%)  10 (38%) | 1(10%)  3 (30%)  6 (60%) | 0.101  0.520  0.337 |
| Worst MGFA classification  　Ⅱ (mild)  　Ⅲ (moderate)  　Ⅳ (severe)  　Ⅴ (crisis) | 1 (4%)  11 (42%)  8 (31%)  6 (23%) | 0 (0%)  2 (20%)  2 (20%)  6 (60%) | 0.876  0.320  0.639  0.094 |
| Worst quantitative MG | 20.7±9.5 | 23.8±5.5 | 0.255 |
| MG-ADL at baseline | 9.2±4.7 | 10.2±5.4 | 0.626 |
| Prednisolone (mg/day) at baseline | 16.2±15.1 | 12.2±5.1 | 0.259 |
| Thymectomy  　Thymectomy for thymoma  　Thymectomy for non-thymoma | 10/10 (100%)  5/16 (31%) | 6/6 (100%)  2/4 (50%) | 1.000  0.617 |
| Fast-acting treatment  　Plasmapheresis  Intravenous immunoglobulin  Intravenous high-dose methylprednisolone | 24 (92%)  24 (92%)  25 (96%) | 10 (100%)  9 (90%)  10 (100%) | 0.741  0.931  0.876 |
| Refractory | 23 (88%) | 10 (100%) | 0.614 |

Supplementary Table S4. Clinical profiles of 15 MG patients treated with ravulizumab.

| No | #14 | #16 | #18 | #19 | #20 | #21 | #22 | #23 | #26 | #29 | #30 | #32 | #34 | #35 | #36 |
| --- | --- | --- | --- | --- | --- | --- | --- | --- | --- | --- | --- | --- | --- | --- | --- |
| Before the switch to ravulizumab |  |  |  |  |  |  |  |  |  |  |  |  |  |  |  |
| MG-ADL | 12 | 9 | 0 | 0 | 5 | 16 | 9 | 7 | 2 | 2 | 13 | 2 | 5 | 5 | 6 |
| MG-Composite scale | 19 | - | 0 | - | 6 | 19 | 14 | - | 3 | 7 | 21 | 4 | 11 | - | 11 |
| MG-QOL15r-J | 13 | - | 6 | 1 | 19 | 25 | 29 | 15 | 1 | 7 | 24 | 9 | 18 | - | 10 |
| Postintervention status | I | U | MM | PR | U | W | I | I | MM | MM | I | I | U | U | I |
| After the treatment with ravulizumab |  |  |  |  |  |  |  |  |  |  |  |  |  |  |  |
| MG-ADL | 17 | 4 | 1 | 1 | 4 | 15 | 7 | 3 | 3 | 2 | 14 | 2 | 2 | 8 | 5 |
| MG-Composite scale | 22 | 6 | 0 | - | 4 | 20 | 10 | - | 4 | 7 | 22 | 4 | 1 | 8 | 11 |
| MG-QOL15r-J | 16 | 23 | 4 | 0 | 12 | 25 | 28 | 15 | 1 | 7 | 27 | 9 | 9 | 21 | 12 |
| Postintervention status | U | I | MM | PR | I | W | I | I | MM | MM | U | I | MM | W | I |
| Reduction of prednisolone (mg/day) | 0 | 0 | 0.5mg | 0 | 0 | 0 | 0 | 0 | 0 | 0 | 0 | 0 | 0 | 0 | 0 |
| EFT during the treatment of ravulizumab | None | IVIg+IVMP | None | None | None | None | None | None | None | None | None | None | None | IVIg+IVMP | None |
| Adverse events | None | None | None | None | None | None | None | None | None | COVID-19 | None | None | None | None | None |

Glossary.

MG: myasthenia gravis, EOMG: early onset MG, LOMG: late onset MG, TAMG: Thymoma associated MG, MGFA: MG Foundation of America, QMG: quantitative MG, IVIg: Intravenous immunoglobulin, IVMP: Intravenous high-dose methylprednisolone, MG-ADL: MG activity of daily living, I: improved, MM: minimal manifestations, U: unchanged, W: worse, PR: pharmacologic remission, EFT: early fast-acting treatment.
