# Supplementary material for: Real‐world experience with eculizumab and switching to ravulizumab for generalized myasthenia gravis
Source: Ann Clin Transl Neurol. 2024 Apr 4;11(5):1338–46. doi: 10.1002/acn3.52051 (PMC11093249; doi:10.1002/acn3.52051)
Supplement: Supplementary file 4 — Figure Captions. [file ACN3-11-1338-s002.docx]

**Figure S1.** Changes in the MG-ADL of #11, #12, and #13 patients. MG-ADL: MG activity of daily living.

**Figure S2.** Changes in the MG-ADL of #5 and #9 patients. MG-ADL: MG activity of daily living.
